# A Family Cluster of Imported Human *Brucella melitensis* Infection with Probable Breast Milk Transmission: A Case Series

**DOI:** 10.3390/tropicalmed10080227

**Published:** 2025-08-14

**Authors:** Christopher Loftus, Jessica Jervis, Victoria Owen, Tom Wingfield, Robert Ball, Waison Wong, Ceri Evans, Christopher Darlow, Francesca Liuzzi, Susan Batley, Rashika Fernando, Alessandro Gerada, Stephen D. Woolley

**Affiliations:** 1 Tropical and Infectious Diseases Unit, University Hospitals of Liverpool Group, Liverpool L7 8XP, UK; 2 Brucella Reference Unit, Liverpool Clinical Laboratories, Liverpool L7 8XP, UK; 3 Liverpool Clinical Laboratories, Department of Microbiology, University Hospital of Liverpool Group, Liverpool L7 8XP, UK; 4 Centre for Tuberculosis Research, Departments of Clinical Sciences and International Public Health, Liverpool School of Tropical Medicine, Liverpool L3 5QA, UK; 5 Department of Global Public Health, Karolinska Institutet, 171 77 Stockholm, Sweden; 6 Department of Paediatric Infectious Diseases and Immunology, Alder Hey Children’s NHS Foundation Trust, Liverpool L14 5AB, UK; 7 Department of Clinical Infection, Microbiology and Immunology, University of Liverpool, Liverpool L69 7BE, UK; 8 Department of Pharmacology and Therapeutics, University of Liverpool, Liverpool L69 7BE, UK; 9 School of Medicine, University of Liverpool, Liverpool L69 7BE, UK; 10 Department of Microbiology, Alder Hey Children’s NHS Foundation Trust, Liverpool L14 5AB, UK; 11 Department of Radiology, University Hospitals of Liverpool Group, Liverpool L7 8XP, UK; 12 Department of Clinical Sciences, Liverpool School of Tropical Medicine, Liverpool L3 5QA, UK

**Keywords:** brucellosis, travel medicine and migrant health, bacteria and bacterial infections

## Abstract

Human brucellosis is a zoonotic, bacterial infection caused by the intracellular, Gram-negative *Brucella* spp., which is common globally but rare in the United Kingdom, with approximately 20 imported cases per annum following travel to countries with high endemicity. Transmission typically occurs via the ingestion of infected animal products, including unpasteurised dairy products. Human-to-human transmission is rare, and routes include postpartum vertical transmission through breastfeeding. We report here on a familial cluster of three cases within a single UK-based Kurdish household of four, including a 11-month-old infant infected through the consumption of breast milk. Four months prior to presentation, the family had travelled together to northern Iraq for a 5-week holiday and all consumed local dairy products except for the children, including the 11-month-old, who was exclusively breastfed at the time. All three patients, including one adult male with complicated brucellosis, had a favourable outcome with medical therapy.: Brucellosis is an important differential diagnosis in returning travellers and specialist advice should be obtained early to prevent sequelae. It is also important for active case-finding, especially in family units with shared exposure. Paediatricians and adult physicians who may manage brucellosis should consider the possibility of vertical transmission in breastfeeding mothers.

## 1. Introduction

Brucellosis, caused by *Brucella* spp. is a common bacterial zoonotic infection globally, with a wide geographical distribution and variable rates of infection between high- and low–middle-income countries [1,2,3,4]. The animal reservoirs for each *Brucella* spp. are linked to a specific animal, with *Brucella melitensis* predominantly being a disease of sheep and goats, *Brucella abortus* infecting cattle, *Brucella suis* biovars 1 to 3 infecting swine, and *Brucella canis* primarily infecting dogs and canids [5]. The disease is seen in both domesticated and wild animals, with the latter hampering control strategies [5]. In humans, this Gram-negative intracellular coccobacillus causes multi-system disease capable of affecting various organs and tissues, which presents challenges in both diagnosis and effective treatment [2,3]. Although potentially lethal in a proportion of complicated cases, the morbidity of brucellosis is more pronounced, with most of the burden being borne by countries and regions with high endemicity, particularly in low-income and rural settings [1,2].

*B. abortus* and *B. melitensis* are endemic in the Mediterranean, Middle East and North Africa region (MENA), India, and Southeast Asia. *B. suis* biovars 1 and 3 are endemic in North America, rural Australia, and Oceanic islands, especially in wild pig and boar populations, with *B. suis* biovar 2 being endemic in Western and Central Europe [1,2,3,6]. *Brucella melitensis* has three biovars, which are globally distributed. In non-endemic regions, where brucellosis has been almost totally eradicated by means of preventative strategies, cases are comparably rarer and typically imported. However, the greater ease in the international movement of infected domesticated animals and people in the modern era cements brucellosis as an important public health hazard worldwide [7].

*Brucella abortus*, *B. suis*, *B. canis*, and *B. melitensis* are the most common causes of brucellosis in humans, with the latter thought to be the most virulent [1,2,3]. *Brucella* spp. is highly contagious and is shed in the excretions of infected animals, including urine, faeces, products of conception, and milk, in which they are capable of prolonged survival [6,7]. The ingestion of unpasteurised milk and dairy products is a key mode of zoonotic transmission. However, any mucocutaneous contact/consumption of infected animal products has the potential to cause infection, thus putting certain groups at risk, including animal handlers such as slaughterhouse workers, vets, farmers and hunters [1,6]. Moreover, *Brucella* spp. is also capable of airborne transmission if aerosolized, even at low infectious doses, presenting a hazard to laboratory and medical staff exposed to clinical samples [8,9]. As such, it is classified as an WHO Hazard Group 3 organism requiring stringent Level 3 biosafety precautions and monitoring [8,9,10]. Additionally, due to the potential for nefarious usage, *Brucella* spp. is designated as a Class B Bioterrorism agent [8,9].

Other modes of transmission reported in the literature include self-inoculation with live animal vaccines (e.g., *B. abortus* RB51); direct inoculation via cuts or abrasions in the skin is rare [11,12,13,14]. Likewise, there are documented but rare cases of human-to-human transmission, including sexual transmission, organ transplantation, blood transfusion, and vertical transmission to the foetus from an infected mother, including via breastfeeding [1,15]. We report here on a familial cluster of three cases within a single, UK-based Kurdish household of four, including a 11-month-old infant infected through the consumption of breast milk.

## 2. Diagnostics

The UK National Brucella Reference Unit (BRU), based in Liverpool, performs serological and molecular testing for *Brucella* species. The serology aspect of testing comprises three screening assays: Brucellacapt^®^ (an immunocapture assay), IgM ELISA, and IgG ELISA (Vircell, Spain). These assays are performed by the BRU in accordance with the manufacturer’s instructions. Samples that are positive at the screening stage are titrated and confirmed by an in-house microagglutination assay (MAG). The BRU use a Brucellacapt^®^ titre cut-off of ≥160 and MAG titre cut-off of ≥160 to determine a positive serological sample. Positive samples are taken for molecular testing. The in-house-developed, UKAS-accredited pan-species PCR has two targets, BCSP31 and IS711, which are common molecular targets shared amongst *Brucella* species. The BRU works closely with the Animal and Plant Health Agency (APHA), who perform culture confirmation on bacterial isolates.

## 3. Case Presentations

### 3.1. Case 1—Patient A

A 43-year-old Kurdish male with no known medical problems presented to the Emergency Department (ED) complaining of fever, rigors, fatigue, right upper quadrant pain, generalised myalgia/arthralgia, and exertional dyspnoea. He had experienced these symptoms on a fortnightly basis for 14 weeks with recovery in between episodes, in addition to losing 9 kg in weight. These symptoms began 3 weeks after returning from a 5-week family holiday to Sulaymaniyah in Northern Iraq, where he visited with his wife and two daughters, aged 2 years and 11 months. The patient was born and raised in Iraq, was well before his holiday, reported no other recent travel, and had lived in the UK since 2003. While visiting friends and relatives in northern Iraq, the patient recalled consuming cheese, milk, and yoghurt provided by his neighbour, and he was unsure if these dairy products were pasteurised.

**Figure 1:**
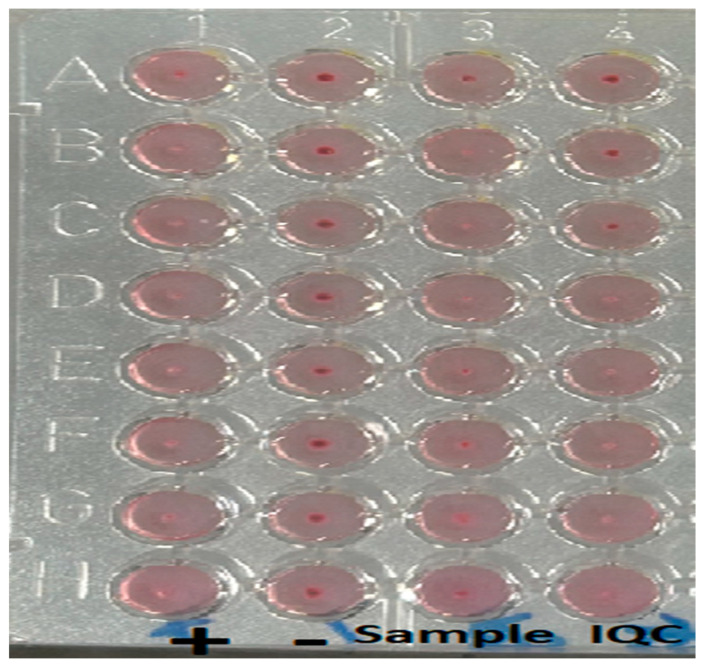
Microagglutination assay plate for Patient A, demonstrating prozone effect. The figure displays a microagglutination plate imaged on a reading mirror following incubation. Samples were incubated for 24 h with safranin and Brucella antigen prior to analysis. The plate is read column-wise, with the highest concentration of the sample being located in row H and the most diluted concentration being located in row A. Column 1 contains the positive control, column 2 contains the negative control, column 3 contains the patient sample, and column 4 contains the internal quality control. Interpretation is based on the appearance of the wells: a red button at the bottom indicates a negative result, while its absence denotes a positive reaction. The patient sample in column 3 demonstrates a prozone effect, initially appearing negative at a 1:20 dilution but turning positive at 1:640 (well 3C), before reverting to negative at 1:2560.

Upon examination in the ED, the patient was afebrile with a tachycardia of 105 beats-per-minute and had right upper quadrant tenderness on abdominal palpation, but there were no concerning bone and joint features. There were no peripheral stigmata of endocarditis or cardiac murmurs. Blood investigations revealed a mildly elevated C-reactive protein (CRP) of 34 mg/L. The patient also had a mild liver transaminitis (alanine transaminase 79 U/L). He was mildly thrombocytopenic (105 × 10^9^/L), microcytic with a mean corpuscular volume (MCV) 79 fL, and had a normal haemoglobin (150 g/L) and normal peripheral white cell count (5.0 × 10^9^/L). The patient was referred to the Infectious Diseases team for review and admitted to hospital. Two sets of peripherally collected blood cultures were positive at 72 h, with Gram-negative coccobacilli observed upon Gram-staining. A preliminary identification was made via matrix-assisted laser desorption/ionisation time of flight assay (MALDI-TOF), which identified the organism as *B. melitensis* (MALDI-TOF score 2.14). A slope of the isolate was sent to the Animal and Plant Health Agency (APHA) for molecular confirmation, with *B. melitensis* biovar 3 confirmed. Serology was also strongly positive, with high BrucellaCapt and MAG titres (Table 1). Interestingly, there was a prozone effect observed with MAG (Figure 1). The pan-Brucella PCR was also positive (BCSP Ct36 and IS711 Ct36).

**Figure 2:**
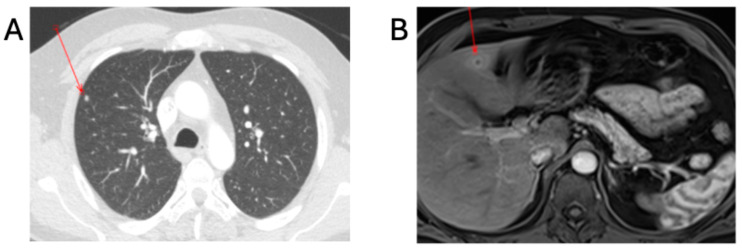
Radiological findings in patient A. (**A**) CT-TAP with contrast showing 5 mm right upper lobe non-specific pulmonary nodule. (**B**) MRI liver with contrast showing 12 mm hepatic abscess in left lobe. The red arrows demonstrate the lesions.

Further investigations to exclude complicated disease consisted of a transthoracic echocardiogram (TTE) and contrast-enhanced computed-tomography scan of the thorax, abdomen and pelvis (CT-TAP). The TTE was negative, and the CT-TAP showed a 6 mm hypodensity within hepatic segment IV, presenting as an abscess and hyper-enhanced gallbladder walls with fat stranding, suggesting cholecystitis; periportal, pericaval, pericardial, and intrapulmonary lymphadenopathy; and two 5 mm pulmonary nodules in the right upper and lower lobes (Figure 2). Combination antibiotic therapy commenced, consisting of intravenous IV gentamicin at 5 mg/kg once a day and 100 mg oral doxycycline twice a day.

The patient clinically improved over the course of a 6-day admission and completed the remaining 4 days of IV gentamicin (10 days total) via outpatient parenteral antimicrobial therapy (OPAT). Doxycycline was continued and prescribed for a provisional duration of 6 weeks. A contrast-enhanced MRI scan of the liver performed confirmed the presence of an abscess measuring 12 mm in hepatic segment IV B. An outpatient MRI of the whole spine did not show any evidence of osteomyelitis/discitis. Although the patient reported feeling better and good compliance with treatment at a follow-up outpatient clinic appointment, as there was evidence of complicated disease, a decision was taken to add oral rifampicin 600 mg once a day alongside oral doxycycline 100 mg twice a day for a further 6 weeks of treatment (total 12 weeks). Repeat serology testing (Table 1) and repeat imaging performed 6 months after commencing treatment showed reducing titres and resolution of the CT findings.

### 3.2. Case 2—Patient B

A 41-year-old Kurdish female married to patient A was investigated by the ID team after she revealed she was suffering from episodic fever, chills, night sweats, fatigue and myalgia/arthralgia; she estimated weight loss between 15 and 20 kg. Symptoms had started 3 weeks after returning from a 5-week holiday to Northern Iraq with her family and, like her husband, she too had consumed the presumed unpasteurised dairy products. The patient was born and raised in Iraq, was well before her holiday, had not travelled anywhere else recently, and had lived in the UK since 2021. The patient had given birth to her youngest daughter 11 months prior and was still actively breastfeeding her infant.

The patient was afebrile at time of review. There was tenderness over the left sacroiliac joint but otherwise her examination was unremarkable. Blood tests showed microcytic anaemia (haemoglobin 110 g/L, MCV 74.9 fL), a normal white blood cell count (4.3 × 10^9^/L), and CRP of 2 mg/L. Liver and kidney biochemistry profiles were unremarkable. Blood cultures taken at initial assessment were negative; however, her *Brucella* serology was strongly positive (Table 1) and consistent with a diagnosis of acute brucellosis. *Brucella* blood PCR was negative. She declined admission as there was no childcare support for her children while her husband was in hospital. She also needed to accompany her 11-month-old, who was also subsequently admitted to the local paediatric centre.

Due to the practical difficulties of arranging an inpatient admission, she was managed in the ambulatory ID clinic. An MRI scan of the lumbar spine and pelvis and TTE revealed no signs of complicated disease. Treatment options were discussed with the patient, who agreed to combination treatment with IV and oral antibiotics. There was a strong suspicion of mother-to-child transmission of *Brucella* via infected breastmilk. The patient was actively breastfeeding at the time, which not only posed an ongoing transmission risk to the infant if set to continue but also had implications regarding the choice of oral agent for her antimicrobial regimen. Doxycycline is contraindicated in breastfeeding as sufficient levels to cause dental enamel staining and deposition in infant bones can be ingested [16]. After appropriate counselling and referral to the specialist breastfeeding nursing teams, the patient agreed to switch to formula milk and stop breastfeeding. The patient was counselled on steps to avoid mastitis upon the sudden cessation of breastfeeding, and the psychological impact of this was acknowledged. This advice included self-expression of breast milk to comfort, and the patient also consented to providing self-expressed breast milk samples before and during the first 7 days of antibiotic treatment. Breast milk was both culture- and PCR-negative for *Brucella* spp. She commenced IV gentamicin 5 mg/kg via OPAT for a total of 14 days alongside oral doxycycline 100 mg twice a day for 6 weeks, which was deemed adequate for uncomplicated disease. The patient reported good compliance and symptomatic improvement during treatment, and this was mirrored by the falling serological titres taken at follow-up.

### 3.3. Case 3—Patient C

The 11-month-old female infant was assessed by the Paediatric Infectious Diseases team following the diagnosis of brucellosis in her parents. She is UK-born without any medical issues and had never travelled internationally before. The infant was 5 months old when she accompanied her family to Iraq and was exclusively breastfeeding during this period, with no reported illnesses during travel. She was asymptomatic on assessment, with no fevers, and gained weight well while still breastfeeding. Her blood tests showed normal white cell count (7.75 × 10^9^/L), haemoglobin (104 g/L), and CRP (<4 mg/L). Her renal function was normal, and liver enzymes were mildly raised (aspartate aminotransferase 78 iu/L and alanine aminotransferase 57 iu/L). Blood cultures taken peripherally were positive at 48 h, revealing an asymptomatic *B. melitensis* bacteraemia. *Brucella* blood PCR was negative, but her serology was strongly positive, indicating acute brucellosis (Table 1).

She was subsequently admitted for further investigations to exclude complicated disease with a whole-body MRI, which showed no evidence of disseminated brucellosis, and a transthoracic echocardiogram excluded endocarditis. The infant was commenced on IV gentamicin 5 mg/kg, which continued via OPAT for a total of 7 days alongside oral co-trimoxazole 240 mg twice daily and oral rifampicin 10 mg/kg twice daily for 6 weeks. The gentamicin course was intended for a duration of 10 days; however, due to intravenous longline issues, the course was stopped after 7 days. Blood cultures were repeated on Day 3 of treatment and were negative. The infant stopped breastfeeding after a week of treatment and transitioned successfully to formula milk. She was monitored closely in outpatient clinics and remained well, without symptoms or complications from treatment. Serology was repeated 3 months following treatment and showed declining antibody titres (Table 1).

### 3.4. Other Family Members—Patient D

A 2-year-old daughter was also assessed by the Paediatric Infectious Diseases team at the same time as her sister. She was born in the UK and had no previous travel or medical issues. The child was well throughout the family holiday and did not consume any local dairy products. During the assessment, she was well, without symptoms or fevers, and was recovering from a recent respiratory viral illness. Her blood tests showed normal white cell count (8.55 × 10^9^/L), haemoglobin (108 g/L), CRP (<4 mg/L), and renal and liver function. Her blood cultures were negative, as was her serological testing. The patient was not investigated further, received no treatment, and remained well during follow-up clinic assessments.

## 4. Discussion

This case series reports on a household cluster of imported brucellosis, with vertical transmission from mother to infant through breast milk. Human brucellosis is an important diagnosis to remain vigilant of, especially in individuals frequently travelling to endemic regions [1]. The UK does not have endemic disease, with all disease cases being imported or transmitted through laboratory/healthcare exposure. It is essential that all family members and travellers accompanying index cases undertake an appropriate risk assessment and investigation if there are concerns regarding brucellosis. There are two further observations that can be made from this case series. Firstly, there is a need to screen extensively for complicated disease, as seen in Patient A with the hepatic lesion and pulmonary nodules, and secondly, a prozone effect can be observed with the MAG assay.

In this family cluster, all family members travelled to a *Brucella* endemic setting and both adults had significant exposure to a known risk factor, i.e., the consumption of unpasteurised dairy products. In contrast to this conventional mode of transmission, the infant daughter was exclusively breastfeeding during the travel period, which strongly suggests vertical transmission as her mode of infection acquisition. It is possible that placental transmission could have occurred, although this seems less likely due to the disease timelines in the mother and infant. Breast milk tested prior to antibiotic treatment was culture- and PCR-negative for *Brucella* at a time in the mother’s illness where she was comparably well, with no bacteraemia. The absence of a positive culture and PCR from patient B’s breast milk is likely due the absence of bacteraemia in the patient, despite longitudinal sampling. Furthermore, there are no standardised culture and PCR methods for breast milk.

Although recognised as an important mode of vertical transmission, less than 10 cases of human breastmilk transmissions have been reported [17,18,19], and even fewer cases of human breast milk bacterial culture positive or were positive upon molecular testing for *Brucella* spp [19,20,21,22]. Transplacental transmission is another recognised modality of vertical transmission between mother and infant [17]. In natural hosts, transmission through breast milk is well recognised in all species [23,24]. A murine model in mice, which are not natural hosts, demonstrated the shedding of *B. melitensis* in 73% of mice experimentally infected with a reference strain and a human isolate [25]. Furthermore, 50% of the pups showed evidence of brucellae in their spleen, demonstrating vertical transmission [25]. The shedding was intermittent, with only 16% of the infected mice being persistent shedders [25]. The bacteria were found in murine milk macrophages and neutrophils [25]. In *B. abortus*, water buffalos mostly shed at low levels; however, 16% were super-spreaders, with milk containing high concentrations of brucellae [25]. *Brucella* spp. infects macrophages and peripheral mononuclear cells (PMNs) [25]. In macrophages, the bacteria survive in Brucella-containing vacuoles, thus preventing elimination through acidification and reactive oxygen and nitrogen species [25]. It is hypothesised that macrophages and PMNs infected with brucellae migrate from blood across the disrupted blood–milk barrier and are subsequently present in milk [25].

The index case (Patient A) had complicated disease and required longer treatment. Whilst liver enzyme derangement is frequently observed in human brucellosis, hepatosplenic abscesses occur in less than 2% of cases and are frequently observed in *B. melitensis* infection [26,27]. These abscesses are often asymptomatic, although they may present with swinging fevers and/or right upper quadrant pain [26,27]. This patient also had a pulmonary nodule, which responded to treatment following serial imaging. Pulmonary involvement of human brucellosis includes pneumonia, pleural effusion, pulmonary nodules, and granulomas [28]. Other recognised complications include bone and joint manifestations (of which sacroiliitis is the most common); genitourinary complications such as orchitis and epididymitis; cardiac complications such as endocarditis; and CNS complications including neurobrucellosis [1,4]. Such complications highlight the need for a careful history and physical examination with directed investigations such as echocardiography and cross-sectional imaging. Treatment regimens and duration are different in uncomplicated and complicated disease; as such, it is imperative to investigate disease type accordingly.

Bacterial culture is the gold standard for diagnosing human brucellosis; however, serology can be used in the absence of a positive culture [15]. Interpretation of Brucella serology can be difficult, especially in individuals from endemic countries and those with previous infection [15]. BRU uses BrucellaCapt, IgG, IgM and the MAG. The Brucellacapt^®^ immunocapture assay detects all total anti-Brucella antibodies, especially non-agglutinating IgA and IgG, and is a sensitive and specific assay. Agglutination assays can exhibit prozone effects, and therefore it is essential that serial dilutions do not obtain a false negative result [15,29,30]. The prozone phenomenon occurs when there is excess antibody, which inhibits the antigen–antibody lattice needed for a visible result, thus generating a false negative result. Repeated serology is essential to confirm the initial serological results.

## 5. Conclusions

Brucellosis is an important differential diagnosis in returning travellers, and specialist advice should be obtained early to prevent the development of complicated disease such as endocarditis, neurobrucellosis, orthopaedic, and rarer hepatosplenic and pulmonary sequelae. Paediatricians and adult physicians who manage brucellosis should consider the possibility of vertical transmission in breastfeeding mothers.

## Figures and Tables

**Table 1:** Summary of investigations.

	Patient							
	A			B		C		D
**Age**	43 years			41 years		11 months		2 years
**Sex**	Male			Female		Female		Female
**Exposure**	Dairy			Dairy		Breast milk only		None
**Symptoms**	Yes			Yes		No		No
**Blood culture**	*Brucella* *melitensis*			Negative		*Brucella* *melitensis*		Negative
**Blood PCR**	Positive			Negative		Negative		Not performed
**Serology**	**D + 0**	**D + 37**	**D + 164**	**D + 0**	**D + 164**	**D + 0**	**D + 157**	**D + 0**
**Brucellacapt** **^®^**	1:2560	1:5120	1:5120	1:5120	1:640	1:5120	1:160	<1:160
**IgM EIA**	>1:2560	1:160	1:40	>1:40	<1:20	1:320	<1:20	<1:20
**IgG EIA**	>1:2560	1:2560	>1:2560	>1:320	1:320	1:320	1:160	<1:20
**MAG**	1:640	1:640	1:160	1:640	1:80	1:320	<1:20	ND

## Data Availability

The original contributions presented in this study are included in the article. Further inquiries can be directed to the corresponding author.
